# Don't forget to turn around: A case of hematochezia and tenesmus driven by anorectal inflammatory cloacogenic polyp

**DOI:** 10.1002/jpr3.12074

**Published:** 2024-04-18

**Authors:** Maria Aguillera, Lacey Miller, Shagun Sharma, Vivian V. Lemus, Meredith Pittman, Thomas Wallach

**Affiliations:** ^1^ Department of Pediatrics SUNY Downstate Health Sciences University Brooklyn USA; ^2^ A. T. Still University School of Osteopathic Medicine Mesa USA; ^3^ Department of Pediatrics, Division of Pediatric Gastroenterology SUNY Downstate Health Sciences University Brooklyn USA; ^4^ Department of Pathology Maimonides Medical Center Brooklyn USA

**Keywords:** colonoscopy, hematochezia, retroflexion

## Abstract

We report a case of a 13‐year‐old male who presented to the Pediatric Gastroenterology clinic with complaints of abdominal pain and frequent stooling, worsened by hematochezia. Despite undergoing endoscopic evaluation twice within a 1‐year period, the diagnosis of an Inflammatory Cloacogenic Polyp (ICP) was only revealed during the second evaluation, in which rectal retroflexion was performed. This case highlights the importance of maintaining the ICP at the anorectal transitional zone as part of the differential diagnosis when evaluating patients with symptoms of distal colitis.

## 1 INTRODUCTION

Chronic symptoms of hematochezia, frequent stooling, and tenesmus raise significant concern for inflammatory bowel disease (IBD). However, some less common conditions can also mimic these symptoms. One example of a condition which can mimic these symptoms is the inflammatory cloacogenic polyp (ICP). ICP is a benign lesion of the anal transitional zone and lower rectum thought to arise as a result of mucosal injury from conditions such as constipation, Crohn's disease, diverticulitis, colorectal tumors, and penetrative trauma to the anus.^1^ Because of their common location at the anorectal junction, these polyps may easily be missed on endoscopic examination, and can be challenging to identify. We present a case of ICP which was initially missed on endoscopic evaluation due to its location in the anal transitional zone. The polyp was identified after representation to clinic with worsening symptoms, including hematochezia, which resulted in a second endoscopic evaluation which included retroflexion.

## 2 CASE DESCRIPTION

**Figure 1:**
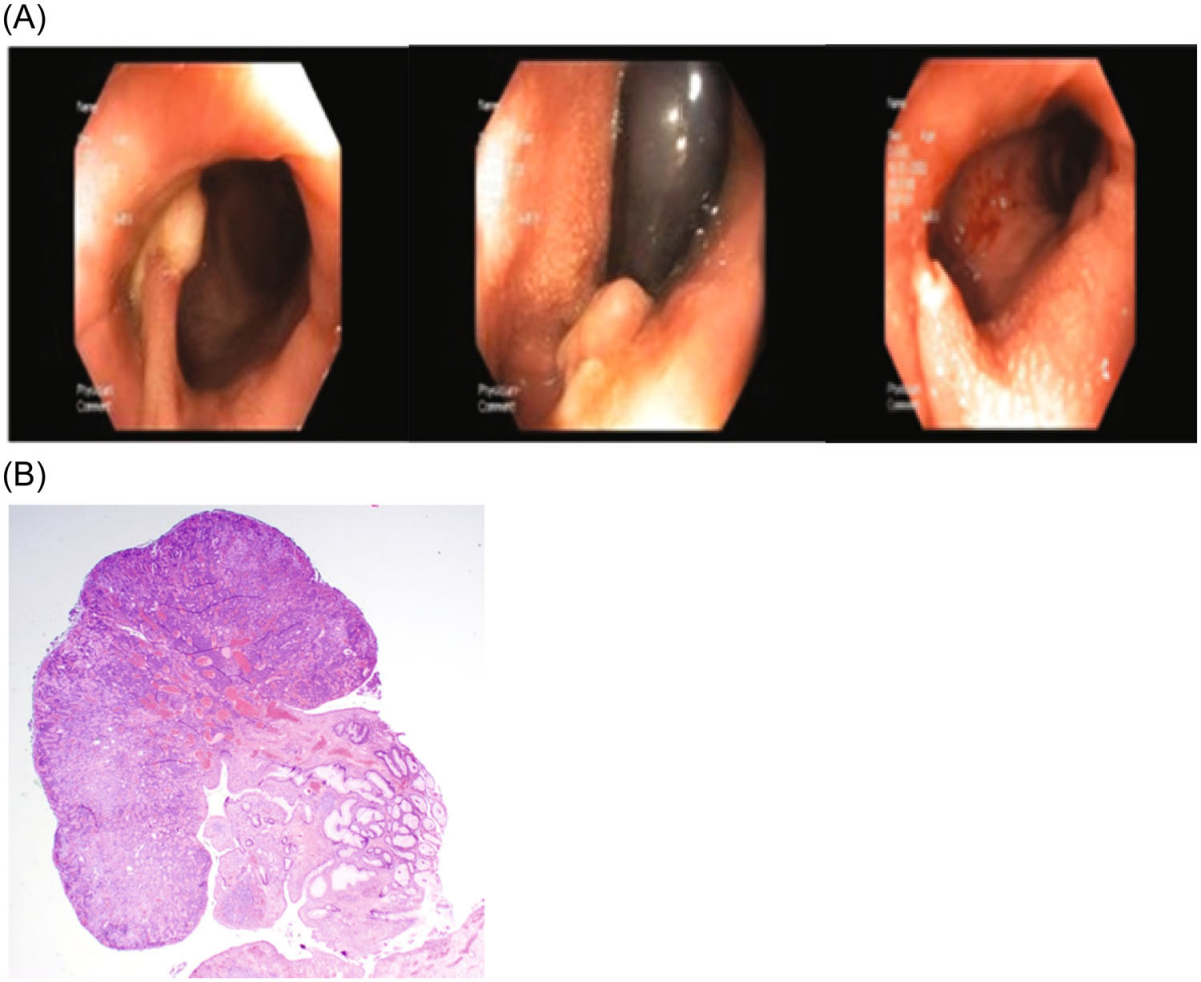
(A) Endoscopic images of the cloacal polyp at diagnosis and after endoscopic removal. (B) Histologic image of cloacal polyp confirming diagnosis.

A 13‐year‐old male with anxiety disorder presented with abdominal pain, frequent bowel movements, and tenesmus. The patient complained of 1 year of colicky, diffuse abdominal pain, frequent stooling, sensation of incomplete emptying, and occasional urgency. At that time, the patient denied hematochezia or melena. Physical exam was notable for epigastric tenderness and hypogastrium fullness with palpable stools. Labs were significant for an elevated erythrocyte sedimentation rate (34 mm/h) with white blood cells elevated to 12.75 K/µL with normal C‐reactive protein and Calprotectin, although patient reported recent viral illness symptoms. A digital rectal exam (DRE) performed in the office revealed no abnormal findings, and no mass was palpated. Esophagogastroduodenoscopy and colonoscopy at the time were unremarkable, with normal histology, and normal magnetic resonance enterography. The patient was diagnosed with irritable bowel syndrome and treated with a combination of ongoing psychiatric care, laxatives, and biofeedback approaches, with substantial improvement in pain. The patient was lost to follow‐up for 1 year but re‐presented with worsened colicky abdominal pain, tenesmus, fecal mucus, sensation of rectal foreign body versus incomplete evacuation, and new hematochezia. The decision to repeat the endoscopic evaluation was made. Repeat DRE at this time was also normal, although did return a small quantity of blood. Repeat colonoscopy was remarkable for the presence of a large (3 cm), partially necrotic polyp located precisely on the anal verge, which was partially removed with a hot snare. Histology showed a polypoid lesion with surface ulceration and underlying dilated tubular colonic crypts in the fibromuscular stroma, consistent with an ICP (Figure 1). The remainder of the evaluation was normal visually and on histology. Symptoms improved but persisted after removal, and patient was referred to pediatric surgery for complete excision of the stalk, with full resolution of symptoms.

## 3 DISCUSSION

The inflammatory cloacogenic polyp was initially described in 1981,^1^ however, most literature on pediatric ICP is limited to case reports.^1^, ^2^, ^3^ ICP can present in different shapes such as ulcerative, polypoidal, and flat polyps with its morphology described as a tubulovillous growth pattern, superficial ulceration, displaced groups of crypts into the submucosa, and inflamed fibromuscular stroma that extends into the lamina propria.^1^, ^4^ The polyp usually contains stratified squamous, transitional, and simple columnar epithelium; surface erosion is often present.^3^ It is associated with thickened and prolapsed muscularis mucosa and is thought to be associated with constipation, rectal prolapse, oncogenic processes, anal trauma, or inflammatory diseases.^2^ ICP is rare in the pediatric population.^2^, ^4^ Clinical symptoms of ICP most often include tenesmus, mucus passage, rectal bleeding, constipation, anal pruritus, and swelling of the anus. Due to its clinical presentation, ICP may be mistaken as IBD. Additionally, fecal calprotectin, a noninvasive marker of gastrointestinal disease, can be elevated with colorectal polyps due to tissue necrosis and active neutrophils. This is further complicated as some have suggested that ICP has the potential to be the initial manifestation of Crohn's disease or connected to the presence of proximal adenocarcinomas.^5^ The overlap in symptoms of IBD and ICP highlights the importance of conducting a colonoscopy with rectal retroflexion (RR), in particular in cases with a concern for colitis which have normal mucosal findings.

ICP is not the only pathology of the anorectal region which can mimic these symptoms. Solitary Rectal Ulcer Syndrome (SRUS) has similar histopathology to the ulcers found in ICP, in fact a biopsy taken from the proximal end of an ICP that does not reveal squamous epithelium of the anal canal may be reported as SRUS.^2^ While SRUS is typically found on the anterior rectal wall 3–10 cm from the anal margin, its underlying mechanism and symptoms are very similar to ICP.^2^ SRUS is theorized to be a result of direct trauma and ischemia, which could be related to the perineum descent and puborectalis contraction during strained defecation, causing compression of the anterior rectal wall, intussusception, and prolapsed rectum.^6^ Symptoms include tenesmus, mucus discharge, rectal pain, changes in bowel habits, and rectal bleeding. SRUS is commonly found in women in the fourth to sixth decade of life.^2^

The utility of routine RR remains somewhat unclear. Adult data suggests an improved adenoma detection rate,^7^ and while it is not extensively studied in pediatrics, Kim et al.^4^ found standardized retroflexion improved detection of multiple lesions, including a substantial improvement in diagnostic yield of ICP.^4^ The anorectal junction is not easily visualized on the colonoscope's straightforward progression and withdrawal, likely contributing to our inability to identify the lesion on the first colonoscopy.^2^, ^3^, ^7^ RR allows for a 360° view of the junction between columnar and cuboidal mucosa, and sometimes it allows visualization of the stratified squamous epithelium. While it is unclear if it is necessary for diagnosis of ICP or SRUS, the work of Kim et al. as well as the challenges inherent in visualizing the anorectal verge and the very distal colon suggest that it may improve diagnostic yield for these conditions. In this case, despite DRE, the lesion was not felt as it was inside the region of pressure exerted by the anal sphincter. Slow advancement of the colonoscope upon entry is suggested and may limit the necessity of rectal retroflection if all walls are observed sufficiently. Nevertheless, these lesions are more likely to be noticed using RR.^2^ While routine retroflection may not be required in every case, RR is safe, has proven increased polyp detection,^4^, ^7^ and can be critical for diagnosing when polyps are not felt on DREs.

## CONFLICT OF INTEREST STATEMENT

The authors declare no conflict of interest.

## ETHICS STATEMENT

Ethical approval obtained from patient and parent at time of second endoscopic evaluation.
